# Estrategias de chinamperos/as para afrontar la inseguridad alimentaria durante la pandemia por COVID-19 en San Gregorio Atlapulco, Ciudad de México, México

**DOI:** 10.1590/0102-311XES131925

**Published:** 2026-03-02

**Authors:** Agustin Pernia, José Alberto Rivera-Márquez, Florence L. Théodore, Laura Elena Corona-de la Peña

**Affiliations:** 1 Universidad Autónoma Metropolitana - Unidad Xochimilco, Ciudad de México, México.; 2 Instituto Nacional de Salud Pública, Cuernavaca, México.; 3 Instituto Nacional de Antropología e Historia, Ciudad de México, México.

**Keywords:** Seguridad Alimentaria, Pueblos Indígenas, COVID-19, Territorio Sociocultural, Organización Social, Food Security, Indigenous Peoples, COVID-19, Sociocultural Territory, Social Organization, Segurança Alimentar, Povos Indígenas, COVID-19, Território Sociocultural, Organização Social

## Abstract

La pandemia por COVID-19 impactó negativamente en la salud-enfermedad y la seguridad alimentaria de los pueblos indígenas de México. Ante esta situación, se organizaron estrategias para hacerle frente a dichas adversidades. Este trabajo se realizó en San Gregorio de Atlapulco, un pueblo al sur de la Ciudad de México y que sostiene una forma prehispánica de producción de alimentos llamada chinampa, con el objetivo de identificar las estrategias que las/los chinamperos/as desarrollaron para afrontar los efectos de la pandemia por COVID-19 en la seguridad alimentaria y la importancia de las chinampas en estas acciones. Se desarrolló una investigación cualitativa mediante entrevistas en profundidad, observación y participación en actividades de organizaciones sociales de chinamperos/as o que trabajan con ellos/as. La información fue analizada mediante análisis de contenido y socializada con los/as participantes. Se identificó que la pandemia provocó la muerte de varios chinamperos/as del pueblo y una reducción de sus ingresos familiares. Esto activó una serie de estrategias de cuidado personal y familiar, pero también vinculaciones con la comunidad y el territorio mediante el intercambio o donación de alimentos y asociaciones con otros/as productores/as chinamperos/as para la comercialización y distribución de sus producciones alimentarias. Lo anterior dio cuenta de la importancia de las chinampas en las condiciones de vida y seguridad alimentaria de esta población ante situaciones de crisis, y del sostenimiento de procesos de autonomía y soberanía sobre el territorio.

## Abstract (en)

The COVID-19 pandemic severely impacted the health and food security of Mexico’s Indigenous peoples, triggering organized community responses. This qualitative study was conducted in San Gregorio Atlapulco, south of Mexico City, home to the pre-Hispanic *chinampa* agricultural system. It aimed to identify the strategies developed by *chinamperos* to mitigate the pandemic’s effects on their food security and analyze the role of *chinamperos* in these actions. Through in-depth interviews, participant observation, and collaboration with *chinampero* social organizations, data were analyzed using content analysis and validated with participants. Findings indicate that the pandemic caused fatalities among *chinamperos* and reduced household incomes. In response, the community activated self-care and familial care strategies, reinforced community and territorial bonds through food exchange and donation and formed partnerships for marketing and distribution. This demonstrates the centrality of *chinampas* in sustaining livelihoods and food sovereignty during crises, underpinning processes of territorial autonomy and self-determination.

## Abstract (pt)

A pandemia da COVID-19 teve um impacto negativo na saúde, nas doenças e na segurança alimentar dos povos indígenas do México. Diante de tal situação, foram organizadas estratégias para enfrentar essas adversidades. Este trabalho foi realizado em San Gregorio de Atlapulco, ao sul da Cidade do México, onde se mantém uma forma pré-hispânica de produção de alimentos chamada *chinampa*, com o objetivo de identificar as estratégias que os *chinamperos* desenvolveram para enfrentar os efeitos da pandemia da COVID-19 na segurança alimentar e a importância das *chinampas* nessas ações. Foi realizada uma pesquisa qualitativa por meio de entrevistas aprofundadas, observação e participação em atividades de organizações sociais de *chinamperos/as* ou que trabalham com eles/as. As informações foram analisadas por meio de análise de conteúdo e compartilhadas com os/as participantes. Identificou-se que a pandemia causou a morte de vários/as *chinamperos/as* da aldeia e uma redução nos rendimentos familiares. Isso ativou uma série de estratégias de cuidados pessoais e familiares, mas também vínculos com a comunidade e com o território por meio da troca ou doação de alimentos, além de parcerias com outros/as *chinamperos/as* para a comercialização e distribuição de suas produções alimentares. O exposto acima demonstrou a importância das chinampas nas condições de vida e segurança alimentar dessa população em situações de crise, bem como na manutenção dos processos de autonomia e soberania sobre o território.

## Introducción

En marzo de 2020, la Organización Mundial de la Salud (OMS) declaró la pandemia por COVID-19. En respuesta, la Secretaría de Salud de México implementó la Jornada Nacional de Sana Distancia para la mitigación y control de riesgos sanitarios, con medidas como el aislamiento voluntario ^1^. No obstante su efectividad, los pueblos indígenas del país enfrentaron mayores niveles de gravedad, hospitalización y muerte por COVID-19 ^2^, debido a la imposibilidad de resguardarse por la necesidad de continuar trabajando, dificultades para acceder a atención médica oportuna, falta de programas sociales específicos y, además, escepticismo frente a la pandemia ^3^, lo que profundizó las desigualdades sociales ya existentes. En 2020, la pobreza moderada y extrema alcanzó al 76,8% de los/as hablantes de una lengua indígena ^4^, mientras que la inseguridad alimentaria escaló al 70,1% de los/as habitantes de zonas rurales ^5^.

La Ciudad de México registró la tasa de mortalidad por COVID-19 más alta del país y, al igual que a nivel nacional, las repercusiones socioeconómicas de la pandemia y del confinamiento tuvieron una distribución desigual. Gran parte de su población debió salir a trabajar debido a la elevada economía informal, especialmente en pueblos y barrios que producen alimentos y los comercializan en la Central de Abastos de la Ciudad de México, principal punto de venta a nivel nacional y uno de los mercados más grandes del mundo ^6^. Un ejemplo es San Gregorio Atlapulco, en la alcaldía Xochimilco, que sostiene una forma prehispánica de producción agrícola denominada chinampa. Los/as chinamperos/as tuvieron que continuar sus actividades pese a las restricciones sanitarias ^6^.

Ante este escenario, se han documentado estrategias que desarrollaron distintos pueblos para mantener sus formas de vida, así como sus vínculos sociales y con la naturaleza durante la emergencia sanitaria ^3^ ^,^ ^7^. Sin embargo, estas estrategias requieren un análisis más profundo, por lo cual el objetivo de este trabajo fue identificar aquellas implementadas por las/los productores/as chinamperos/as de San Gregorio Atlapulco para afrontar los efectos de la pandemia por COVID-19 en la seguridad alimentaria. Asimismo, buscó examinar la relación de dichas estrategias con las chinampas y su compleja articulación entre lo biológico y lo cultural. Para lograrlo, se preguntó: ¿cómo repercutió la pandemia por COVID-19 en la seguridad alimentaria de los/as chinamperos/as? ¿qué estrategias individuales y/o colectivas crearon para afrontar esta situación? ¿cuál fue la importancia de las chinampas en dichas estrategias?

### Contexto de la investigación

San Gregorio Atlapulco es un pueblo de la alcaldía Xochimilco, al sur de la Ciudad de México. Se localiza en la ribera del lago de Xochimilco y tiene una zona lacustre. Cuenta con alrededor de 34 mil habitantes y, pese a su cercanía con la ciudad, mantiene un modo de vida rural urbano ^8^. Algunas viviendas presentan hacinamiento, y los/as pobladores/as enfrentan dificultades para acceder a una vivienda propia. Se identifican 17 asentamientos irregulares que carecen de servicios públicos. Su población se dedica principalmente a la producción de hortalizas, venta ambulante y trabajo doméstico ^8^.

San Gregorio Atlapulco conserva elementos culturales e identitarios prehispánicos, como la producción chinampera ^9^. Según Avendaño García et al. ^10^, una chinampa es un islote artificial creado sobre un lago poco profundo destinado a la siembra. El sistema agrícola chinampero, último vestigio lacustre conservado de la Ciudad de México, es un remanente de los cinco lagos de la Cuenca del Valle de México y abarca las alcaldías de Xochimilco, Tláhuac y Milpa Alta. Se calcula que fue creado alrededor del año 200 a.C. y que, a la llegada de los españoles, existían unas 9,000 hectáreas con capacidad para alimentar a 200.000 personas. Su modo de producción se distingue por el uso de técnicas ancestrales, como el “chapín”, que consiste en la creación de pequeños cuadrados de lodo para la siembra de semillas ^11^.

En San Gregorio Atlapulco, las chinampas aportan al abastecimiento de plantas de ornato y hortalizas a la Ciudad de México mediante la Central de Abastos. A pesar de su gran relevancia para los pobladores, las chinampas enfrentan amenazas como la contaminación ambiental y del agua, el avance de la urbanización y la paulatina pérdida de prácticas culturales ^12^.

El pueblo, previamente fragilizado por el sismo del 19 de septiembre del 2017 (19S), sufrió un impacto significativo en su vida cotidiana. Afectó la productividad de las chinampas y generó pérdidas económicas en las familias. Además, dañó calles, viviendas y edificios públicos, e interrumpió servicios básicos como el suministro de agua y luz. Los/as habitantes denunciaron la falta de coordinación y respuesta por parte del Estado ^10^, lo que generó un antecedente trágico en la historia del pueblo y limitó sus posibilidades de respuesta ante emergencias posteriores, como la pandemia por COVID-19.

### Fundamentación teórica de la investigación

Desde un posicionamiento teórico y metodológico enmarcado en la Medicina Social Latinoamericana y la Epidemiología Crítica ^13^, esta investigación plantea el proceso de alimentación-nutrición como una vía para comprender la interrelación entre ambos fenómenos, al articular dimensiones biológicas y sociales. Dicho proceso es, simultáneamente, expresión y determinante de la salud y la enfermedad, y permite visibilizar los modos históricos -individuales y colectivos- de satisfacer las necesidades alimentarias, así como sus manifestaciones en el estado nutricional ^14^.

En este contexto, producción y consumo constituyen momentos esenciales en la determinación social de los procesos de alimentación-nutrición. En el consumo, las posibilidades del acceso a los alimentos se encuentran condicionadas, entre otros elementos, por la inserción de un grupo social al modo de producción capitalista. Un indicador clave es la seguridad alimentaria, entendida como la satisfacción de las necesidades alimentarias en cantidad y calidad ^14^. Entre las perspectivas sobre esta categoría, se destaca la noción de “seguridad alimentaria tradicional”, que, además de considerar las dimensiones clásicas de disponibilidad, estabilidad, acceso, consumo y utilización biológica, incorpora las formas culturales de aprovisionamiento, como la recolección de alimentos silvestres, el intercambio de semillas y alimentos, y el consumo de alimentos tradicionales ^15^ ^,^ ^16^.

Para analizar la dimensión productiva de la alimentación-nutrición de los/as chinamperos/as, se empleó la categoría “complejo biocultural”, que concibe a la cultura como mediadora entre sociedad y naturaleza para sostener la subsistencia humana en sus dimensiones materiales y simbólicas. Nuestra aproximación se basa en tres vertientes principales: el patrimonio biocultural -espacios de domesticación como las chinampas-, la memoria biocultural -conocimientos tradicionales para la reproducción del territorio y de las estrategias de subsistencia-, y el legado biocultural, que abarca dimensiones agrarias, sociales y espirituales del territorio ^17^. Se retomaron también los aportes críticos sobre la soberanía alimentaria de Micarelli ^18^, comprendida desde una noción de cuidado del territorio, en lugar de control, y reconociendo el sentido de co-pertenencia entre las personas, tierras y alimentos.

Esta investigación propone una relación directa entre la (im)posibilidad de reproducir las chinampas -como complejos bioculturales- y las condiciones de (in)seguridad alimentaria. Partimos de que las estrategias de conservación, individuales y colectivas, generan condiciones para la reproducción social de la comunidad, garantizan su seguridad alimentaria y producen procesos de mayor o menor grado de soberanía alimentaria. Estos vínculos se analizaron durante el confinamiento por COVID-19.

## Material y métodos

Se realizó una investigación cualitativa estructurada en diferentes fases. Su objetivo fue describir las estrategias que, entre marzo y junio de 2020, implementaron los/as chinamperos/as para enfrentar las repercusiones a la seguridad alimentaria durante la pandemia por COVID-19, destacando la importancia de las chinampas en dichas acciones. La generación de información se desarrolló en coordinación con organizaciones civiles con anclaje territorial en Xochimilco (Cuadro 1), que facilitaron el acercamiento al territorio y a productores/as chinamperos/as. En el Cuadro 2 se resumen las características de las técnicas e instrumentos utilizados para la producción de información. El trabajo de campo se desarrolló entre febrero del 2022 y mayo del 2023 y se organizó en tres fases:

**Cuadro 1:** Organizaciones involucradas en el estudio.

NOMBRE DE ORGANIZACIÓN	TIPO DE ORGANIZACIÓN	PRINCIPAL ACTIVIDAD
Cocina CoLaboratorio	Colectivo de académicos/as; agricultores/as; creativos/as; cocineros/as; estudiantes universitarios	Proyecto de incidencia para la creación de nuevas alternativas transdisciplinarias de los sistemas agroalimentarios
Colectivo Zacahuitzco	Colectivo de consumidores y productores	Compra y venta directa de productores/as y comunidades rurales de la Ciudad de México y de los estados vecinos
Redes A.C.	Asociación civil	Conservación y restauración ecológica vinculados al desarrollo social
Granja Apampilco	Cooperativa familiar	Venta de producciones chinamperas y servicios turísticos y de experiencias

Fuente: elaboración propia.

**Cuadro 2:** Características de las técnicas e instrumentos utilizados en la producción de conocimientos.

TÉCNICA	SUJETOS PARTICIPANTES	ESPECIFICIDADES TÉCNICAS
Observación participante	Número de participantes: variable según la actividad, en promedio entre 10 y 20 personas Características: productores/as chinamperos/as de diversas edades, en su mayoría hombres, que asistieron a actividades desarrolladas por una de las organizaciones sociales que participaron en el proyecto. Miembros de la comunidad en los casos de otras observaciones	Número de registros de observación: 14 Aspectos observados: se registraron diálogos, debates y situaciones relacionadas con el territorio, sus prácticas y actores sociales Consideraciones sobre la guía de observación: en el caso de las actividades desarrolladas con una de las organizaciones sociales, se utilizó un modelo de registro proporcionado por ellos/as, el cual contiene una descripción general de la actividad y preguntas disparadoras para la reflexión. En cambio, para las otras observaciones realizadas se utilizó un modelo que consta de una serie de datos introductorios como fecha, lugar y participantes, luego sigue una pequeña introducción que contextualiza la observación, el registro de la actividad propiamente dicha y finaliza con unas breves reflexiones que sirvan para pensar las próximas actividades
Entrevista a profundidad	Número de participantes: 6 Características: 5 varones y una mujer entre 30 y 50 años	Número total de entrevistas: 6 (E1 al E6) Temáticas de la entrevista en profundidad: se incluyeron temáticas sobre el modo de producción chinampero, los conocimientos tradicionales, las festividades comunitarias y las características de las prácticas alimentarias Consideraciones sobre la guía de preguntas: la guía de entrevistas siguió un formato libre de preguntas orientadas por las temáticas anteriormente descriptas. Metodológicamente, se organizó en diferentes momentos temporales: previo, durante y posterior al confinamiento por la pandemia por COVID-19. Se planteó un momento inicial para evocar el primer recuerdo de la chinampa del/la entrevistado/a y recuperar con quién había sido ese momento. Esto permitió dialogar luego sobre las técnicas, conocimientos y rituales de este modo de producción. Asimismo, en este momento se consultó sobre las características de las prácticas alimentarias en la comunidad y en sus familias. Después, se conversó sobre el sismo del 19S, sus repercusiones y estrategias para afrontarlo. Por último, se intercambió sobre la pandemia por COVID-19; sus impactos en el territorio y las estrategias de afrontamiento

Fuente: elaboración propia.

(1) Fase de inmersión (septiembre a noviembre de 2022): aproximación al territorio; sus lógicas, problemáticas y fortalezas, mediante observación participante en actividades desarrolladas por el colectivo Cocina CoLaboratorio. Se produjeron doce registros de campo de talleres y reuniones comunitarias.

(2) Fase de acercamiento inicial con productores/as (diciembre de 2022 y febrero de 2023): permitió el primer diálogo directo con productores/as con el fin de complejizar las categorías del estudio y orientar la siguiente fase. Se realizó una conversación informal con un chinampero; dos entrevistas abiertas y en profundidad con otros dos, y dos registros de campo durante visitas al pueblo, al Mercado de San Gregorio y a una festividad religiosa.

(3) Fase de acercamiento profundo con productores/as (abril a mayo del 2023): ampliación y diversificación de experiencias vinculadas al territorio, la producción y prácticas alimentarias locales, los conocimientos y festividades comunitarias. Se realizaron tres entrevistas abiertas y a profundidad con dos chinamperos y una integrante de una organización social.

Las notas de campo fueron digitalizadas y las entrevistas transcriptas. Posteriormente, se organizó el material y, a través de análisis temático de contenido ^19^, se analizó mediante el siguiente procedimiento:

(1) Categorización inicial: lectura del material e identificación de categorías iniciales.

(2) Sistematización inicial gráfica: organización de categorías preliminares según los momentos: prepandemia, pandemia y postpandemia, y según las dos categorías teóricas expuestas en la fundamentación: complejo biocultural y seguridad alimentaria tradicional, al igual que se identificaron las estrategias de afrontamiento.

(3) Recategorización del material: búsqueda de nuevas categorías y asignación de nuevos nombres a las categorías identificadas en la fase previa.

(4) Resistematización gráfica: reorganización gráfica de las nuevas categorías con el propósito de crear categorías resumen que agruparon a varias subcategorías.

(5) Creación de una matriz de datos: por periodo y objetivo, integrada por nueve libros de Microsoft Excel (https://products.office.com/). En estas matrices, se registró la información categorizada del trabajo de campo.

(6) Elaboración del manual de códigos y conceptualización de categorías y subcategorías: se conceptualizaron los procesos definidos en cada categoría y subcategoría.

La información se trianguló según el tipo de datos proporcionado por la técnica y la fase del trabajo de campo. Los registros de observación se realizaron primero debido a que permitieron documentar las características ecológicas del territorio, identificaron actores, organizaciones e instituciones relevantes, y recopilaron información contextual sobre eventos locales. Posteriormente, estos insumos orientaron el diseño de las entrevistas, posibilitando una exploración detallada de las experiencias y estrategias implementadas para enfrentar las adversidades durante la pandemia.

Además, entre enero y febrero de 2024, se realizó una socialización de resultados con los/as participantes, a través de un proceso de reflexión colectiva y retroalimentación. Como parte del proyecto, elaboramos fanzines y un póster orientado a reflexionar sobre acciones futuras relacionadas con los resultados del estudio. El consentimiento fue otorgado de manera oral. Este estudio fue aprobado por el Comité de Admisión de la Maestría en Medicina Social de la Universidad Autónoma Metropolitana, Unidad Xochimilco, con fecha del 21 de mayo de 2021 (número de oficio MMS.027021). Este trabajo presenta parte de los hallazgos del proyecto de maestría ^20^.

## Resultados

### Las repercusiones de la pandemia por COVID-19 en la salud y procesos de alimentación-nutrición

Según las personas entrevistadas, San Gregorio Atlapulco sufrió un elevado número de contagios por COVID-19. Uno de los productores entrevistados comentó que en el panteón del pueblo contabilizaban entre 5 y 6 muertes al día por esta causa. Adicionalmente, en los registros de observaciones, se identificaron que las expresiones subjetivas individuales en torno a la pandemia se centraron en el miedo a transitar por espacios públicos y, debido a la recomendación oficial de evitar la aglomeración de personas, se registró una falta de ánimos individuales y comunitarios.

Para muchos/as chinamperos/as, la venta en la Central de Abastos -que permaneció abierta- constituía su única fuente de ingresos, lo que los forzó a exponerse al COVID-19, pese al confinamiento, lo que derivó en el contagio y fallecimiento de varios de ellos/as, como se menciona en una de las entrevistas (Cuadro 3). Ello no solo confirió un riesgo laboral adicional, sino también un profundo impacto comunitario: la muerte de los productores amenazó la transmisión de los conocimientos locales sobre las chinampas, mientras que la suspensión de las fiestas comunitarias debilitó los lazos sociales e históricos con el territorio y sus alimentos.

**Cuadro 3:** Fragmentos de las entrevistas a chinamperos/as.

PARTICIPANTE Y FECHA DE LA ENTREVISTA	FRAGMENTO DE LA ENTREVISTA
Las repercusiones de la pandemia por COVID-19 en la salud y procesos de alimentación-nutrición	
Chinampero de San Gregorio Atlapulco, abril de 2023 [E5]	“*Pues de hecho dicen... pues la televisión y los medios de comunicación, ponían a San Gregorio como foco rojo... esta zona de Xochimilco porque como la mayoría de los productores comercializan en la Central de Abastos, por ejemplo, llevo mi papá iba a la Central de Abastos, a lo mejor se contagió allí, un ejemplo, a lo mejor se contagió allá*”
Las estrategias locales para enfrentar la adversidad en la seguridad alimentaria producida por la pandemia por COVID-19	
(a) Estrategias vinculadas a las chinampas como espacios productivos	
Chinampero de San Gregorio Atlapulco, abril de 2023 [E4]	“*...Tengo tres niños: un niño y dos niñas, y mi esposa. Se me enferma mi niña, se me enferma mi esposa. Lo que tuve que hacer es aislarlos y aislar mis dos niños. Entonces en un cuarto de 4x4* [él y su familia vivían en ese cuarto]*, tuve que poner una mica. O sea, dividir, dividirlo, pero como mis niños están pequeños; mi es niña de 5 años y mi niño de 8, yo dije ‘bueno, anímicamente les va a pegar’. O sea, para ellos el COVID era un susto. Entonces dije qué voy a hacer... ya me puse así a pensar rápido, no puedo aislarlos completamente porque los niños van a buscar a su mamá, y mi niña y mi esposa se van a sentir solos. Entonces, qué hago... lo que hice fue poner una mica transparente para que hubiera contacto visual, más no contacto así de persona.* [entonces tuve que] *llegar a separar los trastes, lavar los trastes de los niños aparte, la ropa aparte. Entonces así batallé fácil 20 días*”
(b) Fortalecimiento del tejido socio-territorial mediante estrategias comunitarias	
Chinampero de San Gregorio Atlapulco, abril de 2023 [E4]	“*De hecho, nosotros como productores en algún momento nos organizamos, no muchos, pero llegamos a la Alcaldía con unas cajas de verduras.* [le dijeron a lxs de la Alcaldía] *‘mira esto tú tienes el conocimiento de qué zonas están más afectadas...’ O sea, la idea es que cuando tú haces algo voluntario, no es que tú vayas, te tomes la foto y ahí está. Yo confío en la gente que estaba en la Alcaldía y en algún momento ellos deberían distribuir en las zonas más afectadas, de manera, equitativamente. Porque por desconocimiento, yo podía llegar a una zona y repartirlo, pero esa zona puede ser que no esté muy afectada. Pero como Alcaldía tenía conocimiento, que ellos, la Alcaldía, repartían equitativamente a zonas que estaban en una situación muy lamentable. De hecho, no fueron muchas* [las veces que entregaron cajas con verduras]*, fueron como 2 veces. Pero en lo personal, es algo satisfactorio, el poder colaborar porque el que es humano tiene corazón y el que no, ‘yo me sacudo, a lo mío no le pasó nada y sigo en lo que me importa’*”
Chinampero de San Gregorio Atlapulco, diciembre de 2022 [E2]	Entrevistador: “*¿Compartían con vecinos o vecinas?* [alimentos durante la pandemia]” Chinampero: “*Con vecinos*” Entrevistador: “*¿Con otros chinamperos intercambiaban?*” Chinampero: “*No, con otros chinamperos no. Con vecinos... que vecinos que no producen pues yo les yo les daba este lechugas o lo que tengo de producción yo les daba... y ello pues me daban de otra forma también. No específicamente monetariamente sino en cosas*”
(c) Alianzas territoriales desde el trabajo chinampero	
Chinampero de San Gregorio Atlapulco, abril de 2023 [E4]	Chinampero: “*Sí. Antes de la pandemia, yo trabajaba en el día y en la noche iba a la central...pero cuando después llega todo esto, en algún momento dijéramos por el susto, por lo que estábamos pasando, yo veía diario la información: no pues la Ciudad de México, San Gregorio, es un foco rojo, es el de más contagiados. Dije, bueno ¿qué hago?, tengo producción, qué hago... lo que hice fue: hay un compañero de trabajo que hace varias horas diario... lleva la mercancía. Lo que hicimos fue, ¿sabes qué? Yo te produzco y tú lo vendes*” Entrevistador: “*Pero ¿por acá o hasta la Central?*” Chinampero: “*Hasta la Central.* [Le dijo a su compañero] *¿sabes qué? Te llevas el 5% de las ventas. O sea, porque tienes que girar también para tu familia. Yo no puedo ir porque tengo una familia. O sea, de irme a ganar 1.000 pesos más, después los voy a invertir con mi familia porque se pueden contagiar, porque te expones. O sea, que ya también como que entramos en un dilema, en el que necesitábamos analizar bien las cosas. Te podías ganar 1.000 o 2.000 pesos de ganancia, pero exponías a tu familia completa. Llegabas aquí a las 4 de la mañana tenías que bañarte esas horas, o sea, no, no se puede... Entonces sabes qué hice y yo te mando la mercancía, tú lo vendes y yo no me expongo. Inclusive de ese cambio llevo, después del 19, llevo hasta hoy en día, sigo trabajando igual. Yo produzco y le* [digo] *vamos a ver, que van 20 rollos, van 25 y nomás hacemos cuentas un fin de semana*”
Integrante de cooperativa local, mayo de 2023 [E6]	“*...Sí, pues como te digo que era como nuestra principal fuente de ingresos, vender aquí, el venir al mercado. Entonces tres cooperativas, que ya somos las que tenemos más tiempo como trabajando internamente; cada quien en su cooperativa y en el mercado, que es Olintlalli, nosotros Granja Apampilco y los del pan que se llaman Huautli. Entonces, mi hermana ya tenía, hacía como entregas a domicilio, pero todo era como muy, muy todavía artesanal... muy así y hacía una lista y ponía cuánto iba a entregar porque eran muy poquitas canastas, ¿no? Y cuando nos dicen de la pandemia, que no nos vamos a poder estar en el mercado y así, nos organizamos y les preguntamos a los compañeros que, si quieren venir a entregar a un punto, en una bodeguita, y hacer canastas a domicilio. Nos dicen que pues sí, todos aceptaron y aparte buscamos a otros compañeros de otros mercados que conocíamos, [les preguntaron] que si querían igual hacerlo ¿no? porque sabíamos de la problemática y también porque creíamos que para nuestros clientes sería más atractivo recibir una canasta donde ellos tenían que pagar el costo de envío, donde ya fuera* (...) *todo su mandado*”

Fuente: elaboración propia.

El COVID-19 vulneró la salud y las capacidades de resistencia de los/las chinamperos/as ante la infección; además, el estrés y la angustia derivados de la crisis sociosanitaria exacerbaron las condiciones de salud-enfermedad preexistentes. Adicionalmente, la precariedad de las condiciones materiales de muchos hogares previas a la pandemia incrementó la vulnerabilidad al virus y dificultó el autocuidado. Un ejemplo de ello es el relato de un productor que salía para vender sus productos y quien mencionó que, al vivir en un cuarto con su esposa y sus dos hijos/as, le resultó casi imposible atender las medidas de prevención, lo que derivó en contagios entre ellos. Es importante mencionar que algunas personas se vieron obligadas a vivir la pandemia con familiares, ya que el gobierno aún no terminaba de reconstruir sus viviendas tras el sismo de 19S.

Algunos miembros de la comunidad sostenían que el virus no existía, interpretándolo como parte de una estrategia gubernamental. Esta percepción derivó en la ausencia de prácticas de autocuidado, y el rechazo a la vacunación o al uso de cubrebocas. Tales reacciones pueden entenderse por las históricas relaciones conflictivas del pueblo con distintas instancias del gobierno local, marcadas por disputas previas en torno al agua y la respuesta institucional al sismo.

Quienes sí aceptaron la existencia del COVID-19 activaron prácticas de cuidado en relación con la alimentación-nutrición, ante el riesgo de muerte y el miedo a la pandemia. Los/as productores/as comentaron que intentaron consumir menos productos ultraprocesados y más vegetales de sus chinampas, probablemente como resultado de las conferencias informativas del gobierno sobre los factores de riesgo para desarrollar complicaciones de la enfermedad.

El impacto de la pandemia trascendió los ámbitos de la salud-enfermedad y la alimentación-nutrición. La mayoría de los/as productores/as reconoció que la pandemia afectó sus ingresos y, por tanto, sus condiciones de vida y consumo. Un chinampero manifestó: “*...la pandemia sí nos pegó... y nos pegó fuerte. En primera, porque nosotros tenemos la producción y vamos a comercializar y ahí se paró. Toda la producción que nosotros teníamos no había donde sacarla porque no teníamos compradores*” (entrevista a chinampero de San Gregorio Atlapulco, diciembre de 2022).

Otras consecuencias registradas en las conversaciones observadas entre productores/as fueron la suspensión de paseos turísticos por el lago de Xochimilco; la reducción en las ventas de productos chinamperos debido a la disminución de la movilidad hacia la Central de Abastos; una baja afluencia en mercados locales y el aumento del gasto en salud como consecuencia de enfermedades respiratorias. Este escenario limitó los ingresos de los hogares chinamperos, aunque un productor afín a una de las organizaciones sociales refirió que sus ingresos no solo se mantuvieron, sino que aumentaron, lo cual evidencia diferencias sociales en los impactos de la pandemia, según el tipo de trabajo colaborativo o trabajo individual.

### Las estrategias locales para enfrentar la adversidad en la seguridad alimentaria producida por la pandemia por COVID-19

Ante la adversidad sanitaria, social y simbólica, los/as chinamperos/as y miembros de la comunidad desarrollaron estrategias locales que movilizaron recursos materiales, sociales y simbólicos -disponibles o creados colectivamente- para enfrentar la emergencia y cuidar su salud. Tales estrategias fueron de carácter personal y familiar, comunitarias y de alianza.

#### Estrategias vinculadas a las chinampas como espacios productivos

Se identificaron tres grupos de acciones orientadas a la supervivencia y el cuidado de la salud, orientadas a: (1) preservar la salud y evitar el contagio; (2) compensar la disminución de ingresos producida por la baja de las ventas; y (3) responder a la emergencia ante situaciones inesperadas.

El primero incluyó la reducción de la carga de trabajo chinampero; el retorno de muchos/as productores/as no originarios de la Ciudad de México a sus lugares de origen; vacunación, uso de cubrebocas y el distanciamiento. El Cuadro 3 describe cómo un chinampero tuvo que innovar para cumplir con las recomendaciones oficiales de distanciamiento en su hogar ante un caso positivo. El segundo grupo incluyó el uso de los ahorros familiares para afrontar la reducción de ingresos y el aumento de los gastos en salud, así como un consumo más frecuente de la producción chinampera que, al no venderse, se destinó al autoconsumo y al intercambio. En el tercer grupo se identificó una estrategia relacionada con la muerte repentina de uno/a productor/a, lo que llevó a los familiares a hacerse cargo de las chinampas. Algunos/as continuaron con la producción de alimentos, mientras que otros/as optaron por rentar o vender las chinampas.

Los dos primeros grupos buscaron preservar a los/as productores/as y a las chinampas para sostener el complejo biocultural chinampero pese a la adversidad. El tercer grupo, en cambio, revela dos escenarios opuestos: mientras que conservar las chinampas y mantenerlas productivas permite su reproducción, venderlas o rentarlas conlleva un riesgo de desarticulación del sistema.

#### Fortalecimiento del tejido socio-territorial mediante estrategias comunitarias

El mantenimiento de las prácticas alimentarias y las chinampas, con su articulación bio-cultural compleja, fortaleció el tejido social ante el contexto de crisis. Durante la pandemia, muchas personas manifestaron haber intercambiado con otros miembros de la comunidad su producción chinampera por otros alimentos como: frijol, azúcar y pastas. Frente a los impactos negativos de la pandemia, un grupo de chinamperos/as respondió compartiendo sus cosechas con los barrios y pueblos más afectados (Cuadro 3). Estas iniciativas reforzaron el tejido social local, establecieron formas colectivas de cuidado alimentario y visibilizaron las lógicas comunitarias en torno a la alimentación-nutrición, demostrando cómo en contextos adversos se movilizan recursos solidarios para sostener la vida en común.

Se identificaron, asimismo, estrategias que mantuvieron la articulación biocultural compleja de las chinampas. Por ejemplo, en uno de los registros documentados, un productor relató que, al suspenderse las festividades religiosas, bendijo sus semillas desde su casa durante una misa televisada, en conmemoración del día de la Virgen de la Candelaria, adaptando así una tradición comunitaria a las circunstancias. Esto permitió construir refugios para sostener algunas actividades comunitarias de la cotidianidad, como también cultivar cierta esperanza ante un escenario de profunda incertidumbre. Adicionalmente, personas en teletrabajo, así como algunos/as estudiantes cuyas clases fueron suspendidas por las medidas sociosanitarias, retomaron sus actividades en las chinampas, aunque estas disminuyeron a medida que se retornó a la presencialidad. Estas acciones evidencian cómo las prácticas, conocimientos y cosmovisiones chinamperas permanecen vigentes y resguardadas comunitariamente.

#### Alianzas territoriales desde el trabajo chinampero

Fueron creadas estrategias para enfrentar las repercusiones económicas mediante el establecimiento de alianzas entre actores/as. De menor a mayor nivel de complejidad, se destaca, por ejemplo, la asociación entre dos productores para realizar entregas a la Central de Abastos de la Ciudad de México y, de esta manera, sostener su ingreso económico durante el período más difícil de la pandemia. En el Cuadro 3 se puede encontrar el detalle de esta estrategia.

Entre las estrategias de complejidad intermedia se documentó la asociación con colectivos. Un caso fue el de un productor que, al suspenderse los paseos por el lago de Xochimilco, retomó la producción chinampera junto a su padre, vinculándose con una organización agroecológica chinampera. Este ejemplo ilustra la reactivación de este sistema productivo durante la pandemia, enmarcada en procesos que protegen la articulación biocultural del territorio. Otro productor reportó un incremento en la demanda de sus cosechas, evidenciando la vitalidad de estas redes de apoyo.

Por último, la asociación entre distintas cooperativas que trabajan en un mercado local tuvo una mayor complejidad. El Cuadro 3 muestra que este tipo de estrategias demandó un mayor esfuerzo organizativo, ya que fue necesario coordinar y adaptar “sobre la marcha” las formas más eficientes de comercializar la producción chinampera. Según la persona entrevistada, esto implicó preparar canastas con alimentos y productos para una despensa familiar típica: frutas, verduras, carnes, lácteos, huevos, pan y productos de higiene personal. La entrega estuvo a cargo de otra organización que empleaba bicicletas, una solución práctica ante los problemas de estacionamiento y lo estrecho de algunas vías locales. Adicionalmente, los miembros de las cooperativas productivas asociadas podían adquirir las canastas con un descuento, permitiéndoles generar así un ingreso solidario.

Las estrategias mencionadas contribuyeron a sostener los vínculos entre alimentación, territorio y cultura ante momentos de crisis. Estas requirieron organización y relaciones con otros/as, lo cual, a su vez, produjo cuidados colectivos. En el Cuadro 4 se resumen las estrategias y sus resultados.

**Cuadro 4:** Estrategias desarrolladas por los/as productores/as chinamperos/as para hacer frente a la adversidad producida por la pandemia por COVID-19.

CLASIFICACIÓN	ESTRATEGIA CONCRETA	RESULTADOS CONCRETOS	RELACIONES CON LA COMPLEJIDAD BIOCULTURAL CHINAMPERA
Estrategias personales y familiares	Reducción de la carga del trabajo chinampero	Conservación de la salud personal y familiar	Conservación de la salud de los/as chinamperos/as, quienes tienen el conocimiento chinampero Conservación de la producción chinampera Si se venden o rentan, se desconoce el uso que se le dará al territorio
	Regreso a los pueblos de origen de los/as chinamperos/as foráneos		
	Uso de cubrebocas	Prevención del contagio personal y familiar	
	Vacunación		
	Distanciamiento social		
	Uso de ahorros familiares	Conservación de las fuentes de ingresos	
	Consumo más frecuente de producción chinampera propia		
	Trabajo, renta o venta de las chinampas ante la muerte de un/a productor/a	Atención y resolución de las situaciones de emergencia	
Estrategias comunitarias	Intercambios de alimentos con vecinos/as	Diversificación y complementación de la alimentación Sostenimiento de la trama comunitaria	Sostenimiento de las lógicas comunitarias sobre el territorio Resistencias y negociaciones ante las lógicas del mercado de tierras Conservación del legado de festividades populares bioculturales Reactivación de la memoria biocultural del pueblo Revalorización del territorio por generaciones más jóvenes
	Compartición de producciones chinamperas con zonas más afectadas	Sostenimiento de la trama comunitaria y apoyo solidario	
	Sostenimiento de prácticas de la religiosidad popular en casa	Conservación de los rituales propios de la producción chinampera	
	Regreso al trabajo chinampero por parte de los/as jóvenes y de los/as teletrabajadores/as	Recuperación de la forma cultural de transformación del territorio como producto del trabajo humano	
Estrategias de alianzas	Asociación local con compañeros/as	Sostenimiento de las fuentes de ingreso Creación de formas cooperativas de trabajo	Sostenimiento de las lógicas comunitarias sobre el territorio Contraposición a las lógicas del mercado de tierras Reactivación de la memoria biocultural del pueblo Activación de formas de cuidado colectivo Organización social en torno al territorio socialmente producido
	Asociación de productores/as chinamperos/as con colectivos u organizaciones sociales	Recuperación de la forma cultural de transformación del territorio como producto del trabajo humano Creación o conservación de fuentes de ingresos familiares	
	Cooperación entre organizaciones o cooperativas de productores/as	Conservación de fuentes de ingresos familiares Sostenimientos de formas cooperativas de trabajo	

Fuente: elaboración própria.

## Discusión

En este trabajo se identificaron las repercusiones de la pandemia por COVID-19 tanto en la salud física, como en la subjetividad individual y comunitaria de San Gregorio Atlapulco. Se observó que los chinamperos/as fueron particularmente vulnerables a la pandemia por el antecedente del 19S, debido a la necesidad de seguir trabajando y vendiendo su mercancía, además de tener que vivir con parientes, pese a las restricciones sanitarias. A partir de la experiencia concreta y de los relatos de quienes participaron en este estudio, se identificaron y clasificaron las estrategias en tres tipos: personales y familiares, comunitarias y de alianzas.

La experiencia de los/as chinamperos/as de San Gregorio Atlapulco no es aislada, se inscribe en un patrón de resistencia comunitaria observado en pueblos latinoamericanos que comparten condiciones históricas de desigualdad. Frente a la crisis socio sanitaria, esas comunidades activaron respuestas similares para contrarrestar una adversidad que se caracterizó por: (1) las precarias condiciones de vida preexistentes, (2) una mayor mortalidad por COVID-19 derivada de dicha vulnerabilidad y, (3) la intensificación de los fenómenos de despojo territorial. Surge así la necesidad de contrastar los resultados de esta investigación con experiencias de otros pueblos de México y la región, revelando las bases comunes de su resistencia.

San Gregorio Atlapulco se ubica en una zona de la capital con graves carencias sociales: servicios públicos deficientes, alto grado de informalidad laboral, asentamientos irregulares, hacinamiento y pobreza ^8^ ^,^ ^21^. Esta precariedad estructural se vio exacerbada por el impacto del sismo 19S que mantenía a la comunidad en un proceso de reconstrucción material y simbólica cuando inició la pandemia ^10^, a lo que se sumaba una conflictividad latente con el gobierno local. Esta situación se observó a lo largo de América Latina, donde 80% de la fuerza laboral indígena se emplea en el sector informal, proporción que se eleva aún más entre las mujeres ^22^.

La escasa cobertura y baja resolutividad de los servicios públicos de salud en territorios habitados por poblaciones indígenas incrementó su vulnerabilidad frente al COVID-19. Esto se ha documentado tanto en México ^2^; como en Colombia, Ecuador ^22^ y en Brasil ^23^ ^,^ ^24^, donde la letalidad por COVID-19 fue más elevada entre las poblaciones indígenas que en el resto de la población. En el caso específico de San Gregorio Atlapulco, las actividades productivas de la chinampa aumentaron la exposición y el riesgo de infección entre sus habitantes ^8^.

Más allá de la mayor exposición y mortalidad por COVID-19, la pandemia intensificó diversas amenazas sobre los territorios. En la Ciudad de México se registraron conflictos por la tala clandestina y la extracción de diversos recursos ^25^. En territorios indígenas de Argentina, Chile, Bolivia, Brasil, Perú, Colombia, Nicaragua y El Salvador ^22^ se incrementaron actividades como la expansión minera, la deforestación, la apropiación de fuentes de agua, así como actos de violencia institucional por parte del Estado. En San Gregorio Atlapulco, el tejido social -previamente vulnerado por las disputas hídricas con el gobierno local y los daños del sismo 19S- se fragilizó aún más durante la pandemia. La muerte de chinamperos/as y la consecuente pérdida de sus conocimientos locales, sumado a la suspensión de fiestas comunitarias, pudo haber debilitado las posibilidades de respuesta ante despojos. La gravedad de esta situación quedó al descubierto en diciembre de 2022, cuando el pueblo enfrentó la represión policial de la Ciudad de México por defender sus fuentes de agua ante la construcción de una obra no consultada ^26^.

En este contexto, las estrategias documentadas en San Gregorio Atlapulco pueden situarse como actos de resistencia. Basadas en el territorio chinampero y la comunidad, no solo permitieron enfrentar la adversidad e incertidumbre asociada a la pandemia, sino también proteger su complejidad biocultural. Este hallazgo coincide con investigaciones previas que destacan el papel de las redes alimentarias alternativas para fortalecer los modos de producción tradicional y proteger el conocimiento local ^12^, constituyendo una barrera comunitaria frente al avance de la agroindustria y el efecto debilitante de esta sobre la capacidad colectiva de responder a la crisis.

Diversas comunidades en el país enfrentaron la pandemia mediante estrategias tales como el cierre de entradas a sus territorios, controles de acceso, el fortalecimiento de prácticas de medicina tradicional ^3^, la suspensión de actividades públicas y la prohibición de la venta de productos ultraprocesados ^27^ ^,^ ^28^. Para garantizar la alimentación, comunidades en Morelos y Sonora, al igual que en San Gregorio Atlapulco, incrementaron el consumo de alimentos de sus huertos familiares, la recolección de frutos y la pesca ^3^. En otros casos, para sostener los lazos comunitarios, se emplearon medios digitales para llevar a cabo algunas celebraciones ^3^ y recuperar festividades olvidadas ^28^.

La respuesta de las comunidades indígenas y populares ante la pandemia exhibió una notable capacidad de organización a lo largo de América Latina. En Argentina, la solidaridad se materializó en ollas populares y redes de paisanaje que sostuvieron a los barrios más afectados ^29^. En Brasil, la articulación alcanzó una escala política, con litigios y presión al Estado para hacer efectivos los derechos indígenas ^30^. Mientras, en Ecuador, la juventud desplegó estrategias de comunicación para combatir la desinformación sobre el COVID-19 ^31^. De manera análoga a lo documentado en San Gregorio Atlapulco, comunidades en Colombia implementaron trueques, ventas de canastas a través de redes sociales e incluso un sistema de banderas rojas para identificar y auxiliar a hogares en inseguridad alimentaria ^32^.

## Conclusiones

La pandemia de COVID-19 evidenció y profundizó las inequidades históricas que sufren los pueblos de México y América Latina. Las precarias condiciones de vida y la falta de acceso a servicios de salud colocaron a la población en una situación de alta vulnerabilidad, incrementando su exposición al contagio y a la muerte, comprometiendo su calidad de vida a largo plazo. A ello, se sumó el despojo continuo de sus territorios, configurando así una crisis múltiple donde la emergencia sanitaria amplificó injusticias estructurales preexistentes.

Ante dichas circunstancias, los pueblos latinoamericanos se organizaron mediante estrategias de variada complejidad, que incluyeron:

Conservación de la salud o minimización de los daños por medio de prácticas de cuidado individual y colectivo; 

Mantenimiento o creación de nuevas fuentes de ingresos mediante alianzas, innovaciones y asociaciones;

Autosuficiencia alimentaria comunitaria a través de la producción, pesca y recolección;

Fortalecimiento del tejido comunitario mediante trueques e intercambios de alimentos; sostenimiento y recuperación de festividades populares y comunicación comunitaria, y 

Robustecimiento de la organización social en torno a la defensa del territorio y lucha contra el despojo.

Todas ellas tuvieron un denominador común: el territorio. Esta materialidad, reinterpretada y reproducida históricamente al articular elementos culturales, biológicos y sociales, ha permitido a este sector de la población organizar formas de supervivencia durante la pandemia, lo que demuestra la necesidad de sostener y no obstaculizar procesos autónomos en los territorios; no solo por su importancia en las condiciones de vida, sino en la salud y la alimentación-nutrición. Incluso estas experiencias y estrategias permiten trazar aprendizajes que superan el contexto de pandemia. Rearticular la alimentación con el territorio, la comunidad y los procesos de resistencias ante el despojo constituye una vía para dotar de sentido material y simbólico proyectos societales alternativos para el buen vivir ^33^.

Por lo anterior, es indispensable realizar procesos de investigación en colaboración con las comunidades para identificar, sistematizar y socializar estrategias comunitarias, con el propósito de contribuir, desde los espacios académicos, en la construcción de acervos, dispositivos de resguardo o archivos de memorias colectivas que puedan ser útiles para los pueblos en futuros contextos de crisis. Es importante aclarar que este ejercicio de investigación no debe interpretarse como una forma de eludir las obligaciones del Estado en garantizar las condiciones de vida digna y respeto a la autonomía de los pueblos. Esto es crucial en los actuales contextos de regreso de discursos y políticas de exclusión y discriminación de múltiples sectores oprimidos de la sociedad.

Esta investigación buscó aportar al debate, formas de indagación y socialización de las estrategias de resistencia de los pueblos ante contextos de crisis en un territorio particular. No obstante, es necesario reconocer algunas limitaciones, tales como la cantidad de participantes, lo cual restringió las posibilidades de abordar otras experiencias; la escasa cobertura del territorio xochimilca, San Gregorio Atlapulco solo es uno de los 14 pueblos de Xochimilco. Otras investigaciones podrán recuperar las experiencias de otros pueblos -incluidos los que no son chinamperos- y conocer si las estrategias que aquí se identificaron solo son válidas para afrontar la crisis o crean nuevas formas de organización.

## Data Availability

No aplicable.
